# Supplementary material for: Contralesional Cortical and Network Features Associated with Preoperative Language Deficit in Glioma Patients
Source: Cancers (Basel). 2022 Sep 15;14(18):4469. doi: 10.3390/cancers14184469 (PMC9496725; doi:10.3390/cancers14184469)
Supplement: Supplementary file 1 [file cancers-14-04469-s001.zip › cancers-1867647-supplementary.pdf]

Supplementary materials

Part 1. Supplemental Tables

Table S1. Montreal Neurological Institute (MNI) locations of 123 nodes on the right hemisphere

Regions of interesting	Modified Cyto-architectonic	Right hemisphere		
		X	Y	Z
A10l	lateral BA10	25	61	-4
A10m	medial BA 10	8	58	13
A11l	lateral BA 11	23	36	-18
A11m	medial BA 11	6	57	-16
A12_47l	lateral BA 12/47	42	31	-9
A12_47o	orbital BA 12/47	40	39	-14
A13	BA 13	9	20	-19
A14m	medial BA 14	6	47	-7
A1_2_3ll	BA1/2/3 (lower limb region)	10	-34	54
A1_2_3tonIa	BA 1/2/3 (tongue and larynx region)	56	-10	15
A1_2_3tru	BA1/2/3(trunk region)	20	-33	69
A1_2_3ulhf	BA 1/2/3 (upper limb, head and face region)	50	-14	44
A20cl	caudolateral of BA 20	61	-40	-17
A20cv	caudoventral of BA 20	54	-31	-26
A20il	intermediate lateral BA 20	55	-11	-32
A20iv	intermediate ventral BA 20	46	-14	-33
A20r	rostral BA 20	40	0	-43
A20rv	rostroventral BA 20	33	-15	-34
A21c	caudal BA 21	65	-29	-13
A21r	rostral BA 21	51	6	-32
A22c	caudal BA 22	66	-20	6
A22r	rostral BA 22	56	-12	-5
A23c	caudal BA 23	6	-20	40
A23d	dorsal BA 23	4	-37	32
A23v	ventral BA 23	9	-44	11
A24cd	caudodorsal BA 24	4	6	38
A24rv	rostroventral BA 24	5	22	12
A28_34	BA 28/34 (EC, entorhinal cortex)	19	-10	-30
A2	BA 2	48	-24	48
A31	BA 31 (Lc1)	6	-54	35
A32p	pregenual BA 32	5	28	27
A32sg	subgenual BA 32	5	41	6
A35_36c	caudal BA 35/36	26	-23	-27
A35_36r	rostral BA 35/36	28	-8	-33
A37dl	dorsolateral BA37	60	-53	3
A37elv	extreme lateroventral BA37	53	-52	-18
A37lv	lateroventral BA37	43	-49	-19
A37mv	medioventral BA37	31	-62	-14
A37vl	ventrolateral BA 37	54	-57	-8
A38l	lateral BA 38	47	12	-20
A38m	medial BA 38	31	15	-34
A39c	caudal BA 39(PGp)	45	-71	20
A39rd	rostrodorsal BA 39(Hip3)	39	-65	44
A39rv	rostroventral BA 39(PGa)	53	-54	25
A40c	caudal BA 40(PFm)	57	-44	38
A40rd	rostrodorsal BA 40(PFt)	47	-35	45
A40rv	rostroventral BA 40(PFop)	55	-26	26
A41_42	BA 41/42	54	-24	11
A44d	dorsal BA 44	45	16	25

*BA = Brodmann area.

Table S1. Montreal Neurological Institute coordinates of 123 nodes on the right hemisphere. (continued)

Regions of interesting	Modified Cyto-architectonic	Right hemisphere		
		X	Y	Z
A44op	opercular BA 44	42	22	3
A44v	ventral BA 44	54	14	11
A45c	caudal BA 45	54	24	12
A45r	rostral BA 45	51	36	-1
A46	BA 46	28	55	17
A4hf	BA 4 (head and face region)	55	-2	33
A4ll	BA 4 (lower limb region)	5	-21	61
A4t	BA 4 (trunk region)	15	-22	71
A4tl	BA 4 (tongue and larynx region)	54	4	9
A4ul	BA 4 (upper limb region)	34	-19	59
A5l	lateral BA 5	35	-42	54
A5m	medial BA 5(PEm)	7	-47	58
A6cdl	caudal dorsolateral BA 6	33	-7	57
A6cvl	caudal ventrolateral BA 6	51	7	30
A6dl	dorsolateral BA 6	20	4	64
A6m	medial BA 6	7	-4	60
A6vl	ventrolateral BA 6	34	8	54
A7c	caudal BA 7	19	-69	54
A7ip	intraparietal BA 7(hIP3)	31	-54	53
A7m	medial BA 7(PEp)	6	-65	51
A7pc	postcentral BA 7	23	-43	67
A7r	rostral BA 7	19	-57	65
A8dl	dorsolateral BA 8	22	26	51
A8m	medial BA 8	7	16	54
A8vl	ventrolateral BA 8	42	27	39
A9_46d	dorsal BA 9/46	30	37	36
A9_46v	ventral BA 9/46	42	44	14
A9l	lateral BA 9	13	48	40
A9m	medial BA 9	6	38	35
GP	globus pallidus	22	-2	3
G	hypergranular insula	37	-18	8
IFJ	inferior frontal junction	42	11	39
IFS	inferior frontal sulcus	48	35	13
NAC	nucleus accumbens	15	8	-9
OPC	occipital polar cortex	22	-97	4
Otha	occipital thalamus	13	-27	8
PPtha	posterior parietal thalamus	15	-25	6
Stha	sensory thalamus	18	-22	3
TE1.0_TE1.2	TE1.0 and TE1.2	51	-4	-1
TH	BA TH (medial PPHC)	19	-36	-11
TI	BA TI (temporal agranular insular cortex)	22	1	-36
TL	BA TL (posterior parahippocampal gyrus)	30	-30	-18
V5_MT_plus	BA V5/MT+	48	-70	-1
aSTS	anterior superior temporal sulcus	58	-16	-10
cCunG	caudal cuneus gyrus	8	-90	12
cHipp	caudal hippocampus	29	-27	-10
cLinG	caudal lingual gyrus	10	-85	-9
cTtha	caudal temporal thalamus	10	-14	14
cpSTS	caudoposterior superior temporal sulcus	57	-40	12

*BA = Brodmann area.

Table S1. Montreal Neurological Institute coordinates of 123 nodes on the right hemisphere. (continued)

Regions of interesting	Modified Cyto-architectonic	Right hemisphere		
		X	Y	Z
dCa	dorsal caudate	14	5	14
dIa	dorsal agranular insula	36	18	1
dId	dorsal dysgranular insula	38	5	5
dIg	dorsal granular insula	39	-7	8
dlPu	dorsolateral putamen	29	-3	1
dmPOS	dorsomedial parietooccipital sulcus(PEr)	16	-64	25
iOccG	inferior occipital gyrus	32	-85	-12
lAmyg	lateral amygdala	28	-3	-20
lPFtha	lateral pre-frontal thalamus	13	-16	7
lsOccG	lateral superior occipital gyrus	29	-75	36
mAmyg	medial amygdala	19	-2	-19
mOccG	middle occipital gyrus	34	-86	11
mPFtha	medial pre-frontal thalamus	7	-11	6
mPMtha	pre-motor thalamus	12	-14	1
msOccG	medial superior occipital gyrus	16	-85	34
rCunG	rostral cuneus gyrus	7	-76	11
rHipp	rostral hippocampus	22	-12	-20
rLinG	rostral lingual gyrus	18	-60	-7
rTtha	rostral temporal thalamus	3	-13	5
rpSTS	rostroposterior superior temporal sulcus	53	-37	3
vCa	ventral caudate	15	14	-2
vIa	ventral agranular insula	33	14	-13
vId_vIg	ventral dysgranular and granular insula	39	-2	-9
vmPOS	ventromedial parietooccipital sulcus	15	-63	12
vmPu	rostroventral area 39(PGa)	53	-54	26

*BA = Brodmann area.

Table S2. Nodal efficiencies of all nodes.

Node	Value (Mean ± Standard deviation)			One-way ANOVA (p value)
	AP	mAP	nAP	
A10l_r	0.235 ± 0.023	0.240 ± 0.010	0.242 ± 0.016	0.428
A10m_r	0.185 ± 0.014	0.189 ± 0.010	0.190 ± 0.010	0.385
A11l_r	0.276 ± 0.024	0.284 ± 0.014	0.285 ± 0.014	0.249
A11m_r	0.167 ± 0.087	0.170 ± 0.085	0.188 ± 0.070	0.652
A12_47l_r	0.221 ± 0.024	0.233 ± 0.015	0.242 ± 0.012	**0.002**
A12_47o_r	0.222 ± 0.018	0.232 ± 0.008	0.232 ± 0.010	0.033
A13_r	0.263 ± 0.015	0.267 ± 0.019	0.269 ± 0.009	0.494
A14m_r	0.131 ± 0.054	0.154 ± 0.007	0.136 ± 0.051	0.263
A1_2_3ll_r	0.198 ± 0.012	0.198 ± 0.013	0.207 ± 0.006	0.007
A1_2_3tonIa_r	0.227 ± 0.011	0.227 ± 0.020	0.230 ± 0.013	0.775
A1_2_3tru_r	0.238 ± 0.023	0.243 ± 0.017	0.258 ± 0.011	**0.001**
A1_2_3ulhf_r	0.208 ± 0.008	0.209 ± 0.008	0.216 ± 0.004	**0.001**
A20cl_r	0.226 ± 0.022	0.235 ± 0.013	0.235 ± 0.013	0.213
A20cv_r	0.247 ± 0.009	0.245 ± 0.009	0.250 ± 0.007	0.168
A20il_r	0.256 ± 0.010	0.255 ± 0.012	0.260 ± 0.006	0.205
A20iv_r	0.254 ± 0.007	0.257 ± 0.007	0.260 ± 0.005	0.032
A20r_r	0.277 ± 0.009	0.280 ± 0.010	0.284 ± 0.006	0.048
A20rv_r	0.319 ± 0.012	0.326 ± 0.012	0.328 ± 0.011	0.058
A21c_r	0.239 ± 0.017	0.244 ± 0.016	0.245 ± 0.011	0.361
A21r_r	0.290 ± 0.019	0.296 ± 0.013	0.294 ± 0.012	0.426
A22c_r	0.188 ± 0.017	0.164 ± 0.070	0.190 ± 0.011	0.113
A22r_r	0.226 ± 0.014	0.226 ± 0.012	0.229 ± 0.008	0.498
A23c_r	0.244 ± 0.020	0.251 ± 0.016	0.255 ± 0.010	0.075
A23d_r	0.213 ± 0.013	0.220 ± 0.009	0.220 ± 0.008	0.054
A23v_r	0.217 ± 0.009	0.220 ± 0.015	0.223 ± 0.008	0.235
A24cd_r	0.209 ± 0.015	0.213 ± 0.014	0.218 ± 0.011	0.131
A24rv_r	0.205 ± 0.057	0.223 ± 0.011	0.221 ± 0.017	0.200
A28_34_r	0.208 ± 0.012	0.220 ± 0.011	0.220 ± 0.008	**0.002**
A2_r	0.244 ± 0.011	0.245 ± 0.011	0.254 ± 0.009	**0.003**
A31_r	0.237 ± 0.016	0.246 ± 0.010	0.248 ± 0.008	0.012
A32p_r	0.222 ± 0.024	0.233 ± 0.012	0.232 ± 0.016	0.179
A32sg_r	0.207 ± 0.023	0.215 ± 0.016	0.212 ± 0.018	0.497
A35_36c_r	0.239 ± 0.008	0.241 ± 0.007	0.241 ± 0.009	0.701
A35_36r_r	0.248 ± 0.007	0.252 ± 0.007	0.254 ± 0.006	0.043
A37dl_r	0.227 ± 0.011	0.227 ± 0.012	0.229 ± 0.010	0.857
A37elv_r	0.237 ± 0.011	0.241 ± 0.006	0.243 ± 0.005	0.025
A37lv_r	0.281 ± 0.008	0.282 ± 0.007	0.287 ± 0.005	0.017
A37mv_r	0.267 ± 0.005	0.266 ± 0.011	0.268 ± 0.012	0.827
A37vl_r	0.254 ± 0.012	0.259 ± 0.013	0.261 ± 0.011	0.261
A38l_r	0.241 ± 0.027	0.243 ± 0.013	0.246 ± 0.015	0.686
A38m_r	0.285 ± 0.016	0.294 ± 0.018	0.292 ± 0.014	0.284
A39c_r	0.263 ± 0.011	0.267 ± 0.011	0.269 ± 0.009	0.163
A39rd_r	0.257 ± 0.013	0.260 ± 0.011	0.266 ± 0.012	0.079
A39rv_r	0.242 ± 0.006	0.243 ± 0.009	0.246 ± 0.012	0.464
A40c_r	0.228 ± 0.006	0.228 ± 0.011	0.231 ± 0.009	0.462

*p thershold = 0.004 (FDR corrected)

Table S2. Nodal efficiencies of all nodes. (continued)

Node	Value (Mean ± Standard deviation)			One-way ANOVA (p value)
	AP	mAP	nAP	
A40rd_r	0.264 ± 0.009	0.264 ± 0.011	0.270 ± 0.009	0.094
A40rv_r	0.232 ± 0.019	0.226 ± 0.051	0.240 ± 0.011	0.369
A41_42_r	0.216 ± 0.011	0.205 ± 0.047	0.215 ± 0.013	0.449
A44d_r	0.144 ± 0.053	0.163 ± 0.010	0.167 ± 0.010	0.046
A44op_r	0.232 ± 0.024	0.241 ± 0.018	0.255 ± 0.014	**0.001**
A44v_r	0.092 ± 0.067	0.102 ± 0.059	0.111 ± 0.056	0.625
A45c_r	0.169 ± 0.049	0.147 ± 0.074	0.171 ± 0.064	0.419
A45r_r	0.154 ± 0.064	0.148 ± 0.075	0.187 ± 0.039	0.083
A46_r	0.204 ± 0.057	0.220 ± 0.007	0.215 ± 0.025	0.337
A4hf_r	0.166 ± 0.006	0.159 ± 0.037	0.172 ± 0.003	0.152
A4ll_r	0.215 ± 0.022	0.216 ± 0.022	0.228 ± 0.011	0.033
A4t_r	0.218 ± 0.024	0.224 ± 0.022	0.237 ± 0.014	0.009
A4tl_r	0.222 ± 0.014	0.219 ± 0.027	0.222 ± 0.019	0.880
A4ul_r	0.234 ± 0.013	0.237 ± 0.013	0.248 ± 0.007	**0.001**
A5l_r	0.227 ± 0.009	0.228 ± 0.009	0.234 ± 0.005	0.005
A5m_r	0.200 ± 0.014	0.205 ± 0.014	0.211 ± 0.009	0.034
A6cdl_r	0.224 ± 0.015	0.227 ± 0.014	0.236 ± 0.008	0.009
A6cvl_r	0.141 ± 0.052	0.159 ± 0.009	0.164 ± 0.010	0.045
A6dl_r	0.239 ± 0.025	0.241 ± 0.027	0.255 ± 0.019	0.055
A6m_r	0.226 ± 0.025	0.227 ± 0.025	0.240 ± 0.014	0.049
A6vl_r	0.217 ± 0.017	0.218 ± 0.016	0.229 ± 0.011	0.018
A7c_r	0.216 ± 0.012	0.216 ± 0.012	0.223 ± 0.006	0.052
A7ip_r	0.233 ± 0.010	0.232 ± 0.008	0.240 ± 0.005	0.003
A7m_r	0.158 ± 0.065	0.160 ± 0.067	0.152 ± 0.076	0.921
A7pc_r	0.210 ± 0.016	0.211 ± 0.015	0.220 ± 0.014	0.090
A7r_r	0.185 ± 0.052	0.173 ± 0.059	0.204 ± 0.015	0.077
A8dl_r	0.212 ± 0.017	0.215 ± 0.014	0.224 ± 0.010	0.016
A8m_r	0.205 ± 0.015	0.208 ± 0.017	0.213 ± 0.011	0.230
A8vl_r	0.185 ± 0.020	0.191 ± 0.007	0.191 ± 0.009	0.247
A9_46d_r	0.207 ± 0.020	0.214 ± 0.009	0.215 ± 0.012	0.215
A9_46v_r	0.176 ± 0.048	0.188 ± 0.011	0.185 ± 0.017	0.386
A9l_r	0.188 ± 0.053	0.203 ± 0.011	0.204 ± 0.015	0.186
A9m_r	0.196 ± 0.013	0.199 ± 0.009	0.203 ± 0.008	0.076
GP_r	0.191 ± 0.031	0.185 ± 0.063	0.209 ± 0.043	0.249
G_r	0.214 ± 0.016	0.218 ± 0.014	0.216 ± 0.020	0.765
IFJ_r	0.172 ± 0.065	0.194 ± 0.014	0.199 ± 0.015	0.056
IFS_r	0.142 ± 0.041	0.153 ± 0.007	0.151 ± 0.012	0.343
NAC_r	0.257 ± 0.017	0.267 ± 0.009	0.269 ± 0.009	0.011
OPC_r	0.226 ± 0.006	0.226 ± 0.006	0.229 ± 0.007	0.158
Otha_r	0.213 ± 0.009	0.213 ± 0.009	0.208 ± 0.043	0.820
PPtha_r	0.243 ± 0.013	0.245 ± 0.012	0.250 ± 0.013	0.191
Stha_r	0.173 ± 0.047	0.184 ± 0.017	0.189 ± 0.014	0.171
TE1.0_TE1.2_r	0.238 ± 0.010	0.236 ± 0.012	0.237 ± 0.012	0.840
TH_r	0.245 ± 0.013	0.248 ± 0.010	0.247 ± 0.012	0.830
TI_r	0.249 ± 0.018	0.253 ± 0.016	0.256 ± 0.012	0.355

*p thershold = 0.004 (FDR corrected)

Table S2. Nodal efficiencies of all nodes. (continued)

Node	Value (Mean ± Standard deviation)			One-way ANOVA (p value)
	AP	mAP	nAP	
TL_r	0.244 ± 0.007	0.245 ± 0.010	0.248 ± 0.006	0.186
V5_MT_plus_r	0.257 ± 0.009	0.259 ± 0.009	0.264 ± 0.005	0.019
aSTS_r	0.239 ± 0.019	0.251 ± 0.008	0.251 ± 0.010	0.013
cCunG_r	0.221 ± 0.007	0.220 ± 0.009	0.223 ± 0.010	0.499
cHipp_r	0.261 ± 0.012	0.266 ± 0.010	0.270 ± 0.008	0.021
cLinG_r	0.228 ± 0.011	0.229 ± 0.015	0.235 ± 0.009	0.148
cTtha_r	0.208 ± 0.018	0.218 ± 0.011	0.219 ± 0.013	0.057
cpSTS_r	0.209 ± 0.008	0.211 ± 0.010	0.211 ± 0.012	0.693
dCa_r	0.226 ± 0.011	0.227 ± 0.015	0.219 ± 0.017	0.593
dIa_r	0.167 ± 0.049	0.185 ± 0.012	0.194 ± 0.009	0.010
dId_r	0.207 ± 0.015	0.211 ± 0.016	0.215 ± 0.017	0.347
dIg_r	0.177 ± 0.007	0.169 ± 0.040	0.159 ± 0.059	0.484
dlPu_r	0.242 ± 0.033	0.254 ± 0.019	0.270 ± 0.015	**0.001**
dmPOS_r	0.241 ± 0.009	0.247 ± 0.006	0.249 ± 0.005	**0.003**
iOccG_r	0.252 ± 0.006	0.252 ± 0.009	0.256 ± 0.008	0.153
lAmyg_r	0.216 ± 0.063	0.239 ± 0.016	0.242 ± 0.015	0.056
lPFtha_r	0.221 ± 0.059	0.233 ± 0.025	0.241 ± 0.022	0.248
lsOccG_r	0.246 ± 0.013	0.250 ± 0.012	0.254 ± 0.007	0.053
mAmyg_r	0.234 ± 0.018	0.237 ± 0.015	0.239 ± 0.013	0.617
mOccG_r	0.253 ± 0.009	0.256 ± 0.008	0.258 ± 0.005	0.071
mPFtha_r	0.230 ± 0.018	0.232 ± 0.018	0.238 ± 0.019	0.383
mPMtha_r	0.162 ± 0.064	0.149 ± 0.076	0.186 ± 0.019	0.092
msOccG_r	0.248 ± 0.010	0.252 ± 0.008	0.256 ± 0.005	0.016
rCunG_r	0.223 ± 0.012	0.231 ± 0.008	0.230 ± 0.010	0.080
rHipp_r	0.267 ± 0.014	0.272 ± 0.013	0.277 ± 0.012	0.047
rLinG_r	0.264 ± 0.016	0.273 ± 0.016	0.275 ± 0.013	0.075
rTtha_r	0.179 ± 0.052	0.196 ± 0.017	0.198 ± 0.015	0.130
rpSTS_r	0.211 ± 0.019	0.219 ± 0.010	0.216 ± 0.023	0.388
vCa_r	0.258 ± 0.027	0.268 ± 0.012	0.271 ± 0.015	0.112
vIa_r	0.224 ± 0.032	0.231 ± 0.033	0.243 ± 0.020	0.131
vId_vIg_r	0.187 ± 0.056	0.196 ± 0.050	0.198 ± 0.045	0.794
vmPOS_r	0.251 ± 0.009	0.256 ± 0.010	0.259 ± 0.007	0.043
vmPu_r	0.212 ± 0.042	0.226 ± 0.053	0.238 ± 0.049	0.265

*p thershold = 0.004 (FDR corrected)

Table S3. Degree Centralities of all nodes.

Node	Value (Mean ± Standard deviation)			One-way ANOVA (p value)
	AP	mAP	nAP	
A10l_r	2.733 ± 0.573	2.700 ± 0.458	2.640 ± 0.557	0.859
A10m_r	2.867 ± 0.340	2.950 ± 0.218	2.880 ± 0.325	0.665
A11l_r	6.400 ± 0.879	6.400 ± 0.860	6.480 ± 0.700	0.934
A11m_r	0.800 ± 0.400	0.800 ± 0.400	0.880 ± 0.325	0.725
A12_47l_r	2.533 ± 0.618	2.900 ± 0.300	2.880 ± 0.325	0.024
A12_47o_r	1.867 ± 0.340	2.000 ± 0.000	1.920 ± 0.271	0.287
A13_r	3.733 ± 0.442	3.650 ± 0.654	3.640 ± 0.480	0.862
A14m_r	0.867 ± 0.340	1.000 ± 0.000	0.880 ± 0.325	0.263
A1_2_3ll_r	2.933 ± 0.249	2.850 ± 0.357	2.960 ± 0.196	0.413
A1_2_3tonIa_r	3.533 ± 0.499	3.650 ± 0.726	3.600 ± 0.632	0.872
A1_2_3tru_r	4.400 ± 1.020	4.400 ± 0.663	4.720 ± 0.531	0.266
A1_2_3ulhf_r	3.000 ± 0.000	2.950 ± 0.218	3.000 ± 0.000	0.374
A20cl_r	3.400 ± 0.800	3.750 ± 0.536	3.680 ± 0.546	0.248
A20cv_r	4.733 ± 0.573	4.650 ± 0.654	4.840 ± 0.463	0.544
A20il_r	4.800 ± 0.400	4.500 ± 0.806	4.760 ± 0.427	0.241
A20iv_r	3.933 ± 0.249	3.900 ± 0.300	3.960 ± 0.196	0.736
A20r_r	4.867 ± 0.340	4.700 ± 0.557	4.880 ± 0.325	0.338
A20rv_r	11.867 ± 0.957	12.300 ± 0.900	12.120 ± 1.032	0.449
A21c_r	3.400 ± 0.611	3.550 ± 0.805	3.600 ± 0.490	0.643
A21r_r	6.800 ± 1.470	7.350 ± 0.853	6.960 ± 1.038	0.329
A22c_r	1.600 ± 0.490	1.500 ± 0.742	1.560 ± 0.496	0.884
A22r_r	4.067 ± 1.062	3.950 ± 0.921	4.280 ± 0.601	0.438
A23c_r	7.800 ± 1.222	8.450 ± 0.805	7.960 ± 0.916	0.124
A23d_r	2.733 ± 0.442	3.000 ± 0.000	2.880 ± 0.325	0.052
A23v_r	2.867 ± 0.340	2.800 ± 0.510	2.880 ± 0.325	0.797
A24cd_r	3.333 ± 0.789	3.750 ± 0.433	3.520 ± 0.574	0.139
A24rv_r	1.800 ± 0.542	1.900 ± 0.300	1.880 ± 0.325	0.741
A28_34_r	2.133 ± 0.718	2.650 ± 0.572	2.520 ± 0.574	0.055
A2_r	4.600 ± 0.611	4.600 ± 0.583	4.840 ± 0.463	0.268
A31_r	5.000 ± 0.730	5.650 ± 0.726	5.520 ± 0.640	0.026
A32p_r	4.267 ± 0.929	4.700 ± 0.557	4.520 ± 0.640	0.219
A32sg_r	2.733 ± 0.442	2.800 ± 0.510	2.760 ± 0.427	0.914
A35_36c_r	3.667 ± 0.699	3.850 ± 0.357	3.600 ± 0.632	0.365
A35_36r_r	4.333 ± 0.699	4.500 ± 0.671	4.280 ± 0.531	0.509
A37dl_r	2.800 ± 0.400	2.650 ± 0.477	2.720 ± 0.449	0.634
A37elv_r	3.667 ± 0.596	3.950 ± 0.218	3.960 ± 0.196	0.031
A37lv_r	6.733 ± 0.442	6.550 ± 0.497	6.880 ± 0.325	0.045
A37mv_r	4.000 ± 0.000	3.900 ± 0.300	3.880 ± 0.325	0.404
A37vl_r	4.867 ± 1.024	5.450 ± 0.805	5.320 ± 0.835	0.154
A38l_r	3.333 ± 0.943	3.100 ± 0.768	3.480 ± 0.700	0.301
A38m_r	5.933 ± 0.772	5.900 ± 1.221	5.720 ± 0.873	0.760
A39c_r	6.133 ± 0.884	6.300 ± 0.843	6.440 ± 0.637	0.499
A39rd_r	5.333 ± 0.789	5.350 ± 0.654	5.720 ± 0.722	0.157
A39rv_r	4.733 ± 0.442	4.750 ± 0.536	4.880 ± 0.431	0.557
A40c_r	2.667 ± 0.471	2.650 ± 0.572	2.760 ± 0.427	0.735

*p thershold = 0.004 (FDR corrected)

Table S3. Degree Centralities of all nodes. (continued)

Node	Value (Mean ± Standard deviation)			One-way ANOVA (*p* value)
	AP	mAP	nAP	
A40rd_r	6.533 ± 0.618	6.600 ± 0.663	6.680 ± 0.546	0.763
A40rv_r	4.067 ± 0.854	4.250 ± 0.942	4.440 ± 0.753	0.416
A41_42_r	2.867 ± 0.340	2.700 ± 0.640	2.560 ± 0.637	0.291
A44d_r	2.667 ± 0.471	2.750 ± 0.433	2.800 ± 0.400	0.654
A44op_r	5.400 ± 0.712	5.400 ± 0.583	5.760 ± 0.427	0.067
A44v_r	0.667 ± 0.471	0.750 ± 0.433	0.800 ± 0.400	0.654
A45c_r	0.933 ± 0.249	0.800 ± 0.400	0.880 ± 0.325	0.514
A45r_r	0.867 ± 0.340	0.800 ± 0.400	0.960 ± 0.196	0.254
A46_r	3.533 ± 0.806	3.850 ± 0.357	3.520 ± 0.700	0.203
A4hf_r	1.000 ± 0.000	0.950 ± 0.218	1.000 ± 0.000	0.374
A4ll_r	3.533 ± 0.499	3.350 ± 0.654	3.600 ± 0.566	0.372
A4t_r	3.067 ± 0.998	3.300 ± 0.900	3.640 ± 0.625	0.110
A4tl_r	2.800 ± 0.400	2.750 ± 0.622	2.640 ± 0.557	0.649
A4ul_r	4.533 ± 0.499	4.700 ± 0.557	4.840 ± 0.367	0.158
A5l_r	3.867 ± 0.340	3.850 ± 0.477	3.920 ± 0.271	0.811
A5m_r	2.667 ± 0.471	2.750 ± 0.433	2.840 ± 0.367	0.458
A6cdl_r	3.733 ± 0.442	3.700 ± 0.557	3.720 ± 0.449	0.980
A6cvl_r	2.000 ± 0.000	2.000 ± 0.000	2.000 ± 0.000	1.000
A6dl_r	4.467 ± 0.718	4.300 ± 1.005	4.680 ± 0.546	0.278
A6m_r	4.400 ± 0.712	4.450 ± 0.589	4.520 ± 0.574	0.836
A6vl_r	3.667 ± 0.471	3.550 ± 0.805	3.800 ± 0.490	0.414
A7c_r	2.733 ± 0.573	2.550 ± 0.669	2.760 ± 0.427	0.439
A7ip_r	4.667 ± 0.596	4.450 ± 0.669	4.720 ± 0.449	0.289
A7m_r	0.867 ± 0.340	0.850 ± 0.357	0.800 ± 0.400	0.842
A7pc_r	2.667 ± 0.596	2.550 ± 0.589	2.800 ± 0.400	0.301
A7r_r	1.667 ± 0.596	1.450 ± 0.669	1.880 ± 0.325	0.038
A8dl_r	3.667 ± 0.471	3.600 ± 0.583	3.960 ± 0.196	0.020
A8m_r	3.400 ± 0.611	3.700 ± 0.458	3.360 ± 0.625	0.135
A8vl_r	2.733 ± 0.680	2.950 ± 0.218	2.840 ± 0.367	0.363
A9_46d_r	3.667 ± 0.596	4.000 ± 0.000	3.880 ± 0.325	0.039
A9_46v_r	2.733 ± 0.573	2.900 ± 0.300	2.760 ± 0.427	0.466
A9l_r	2.400 ± 0.800	2.800 ± 0.400	2.680 ± 0.546	0.147
A9m_r	3.000 ± 0.000	3.000 ± 0.000	3.000 ± 0.000	1.000
GP_r	1.733 ± 0.442	1.550 ± 0.589	1.800 ± 0.400	0.238
G_r	2.467 ± 0.618	2.650 ± 0.477	2.520 ± 0.640	0.638
IFJ_r	3.600 ± 0.712	3.800 ± 0.400	3.800 ± 0.400	0.426
IFS_r	0.933 ± 0.249	1.000 ± 0.000	1.000 ± 0.000	0.226
NAC_r	4.600 ± 0.611	4.800 ± 0.400	4.800 ± 0.400	0.374
OPC_r	3.733 ± 0.442	3.600 ± 0.583	3.800 ± 0.490	0.448
Otha_r	2.867 ± 0.340	2.600 ± 0.663	2.400 ± 0.800	0.120
PPtha_r	5.400 ± 0.879	5.250 ± 0.829	5.400 ± 0.894	0.829
Stha_r	1.867 ± 0.340	1.700 ± 0.458	1.800 ± 0.400	0.492
TE1.0_TE1.2_r	4.800 ± 0.400	4.450 ± 0.589	4.440 ± 0.697	0.161
TH_r	3.667 ± 0.596	3.700 ± 0.458	3.400 ± 0.566	0.152
TI_r	3.533 ± 0.806	3.400 ± 0.735	3.520 ± 0.700	0.834

*p thershold = 0.004 (FDR corrected)

Table S3. Degree Centralities of all nodes. (continued)

Node	Value (Mean ± Standard deviation)			One-way ANOVA (*p* value)
	AP	mAP	nAP	
TL_r	3.600 ± 0.490	3.600 ± 0.735	3.720 ± 0.531	0.757
V5_MT_plus_r	4.533 ± 0.618	4.550 ± 0.669	4.680 ± 0.466	0.678
aSTS_r	4.133 ± 0.806	4.450 ± 0.497	4.560 ± 0.804	0.209
cCunG_r	3.867 ± 0.340	3.650 ± 0.572	3.720 ± 0.531	0.471
cHipp_r	5.400 ± 0.712	5.550 ± 0.740	5.600 ± 0.566	0.665
cLinG_r	3.533 ± 0.618	3.350 ± 0.792	3.760 ± 0.427	0.104
cTtha_r	3.267 ± 0.772	3.550 ± 0.589	3.200 ± 0.849	0.303
cpSTS_r	2.000 ± 0.000	1.950 ± 0.218	2.000 ± 0.000	0.374
dCa_r	0.000 ± 0.000	0.000 ± 0.000	0.000 ± 0.000	1.000
dIa_r	0.933 ± 0.249	1.000 ± 0.000	1.000 ± 0.000	0.226
dId_r	1.933 ± 0.249	2.000 ± 0.000	1.920 ± 0.271	0.460
dIg_r	1.000 ± 0.000	0.950 ± 0.218	0.880 ± 0.325	0.328
dlPu_r	4.067 ± 0.929	4.050 ± 0.921	4.520 ± 0.640	0.118
dmPOS_r	4.000 ± 0.000	3.850 ± 0.357	4.000 ± 0.000	0.042
iOccG_r	5.533 ± 0.499	5.300 ± 0.781	5.560 ± 0.697	0.432
lAmyg_r	2.467 ± 0.806	2.750 ± 0.433	2.800 ± 0.400	0.171
lPFtha_r	4.400 ± 0.879	4.350 ± 0.853	4.520 ± 0.806	0.795
lsOccG_r	4.267 ± 0.772	4.550 ± 0.805	4.760 ± 0.427	0.094
mAmyg_r	2.733 ± 0.442	2.700 ± 0.458	2.720 ± 0.449	0.977
mOccG_r	5.267 ± 0.772	5.500 ± 0.671	5.520 ± 0.574	0.483
mPFtha_r	3.467 ± 0.806	3.450 ± 0.589	3.480 ± 0.755	0.991
mPMtha_r	1.867 ± 0.499	1.450 ± 0.805	1.680 ± 0.466	0.149
msOccG_r	4.467 ± 0.499	4.300 ± 0.640	4.600 ± 0.490	0.213
rCunG_r	2.933 ± 0.249	3.000 ± 0.000	2.920 ± 0.271	0.460
rHipp_r	6.067 ± 0.772	6.550 ± 0.669	6.720 ± 0.531	0.013
rLinG_r	5.400 ± 0.611	5.650 ± 0.726	5.640 ± 0.625	0.481
rTtha_r	1.600 ± 0.611	1.750 ± 0.433	1.680 ± 0.466	0.691
rpSTS_r	2.467 ± 0.618	2.750 ± 0.433	2.560 ± 0.637	0.341
vCa_r	5.000 ± 0.966	5.200 ± 0.980	5.240 ± 0.950	0.747
vIa_r	2.400 ± 0.712	2.450 ± 0.669	2.640 ± 0.557	0.459
vId_vIg_r	1.667 ± 0.596	1.750 ± 0.536	1.640 ± 0.557	0.810
vmPOS_r	4.933 ± 0.249	4.850 ± 0.357	4.880 ± 0.325	0.757
vmPu_r	2.467 ± 0.618	2.650 ± 0.654	2.880 ± 0.431	0.089

*p thershold = 0.004 (FDR corrected)

Table S4. Regions with elevated cortical volume identified by VBM.

contrasts	Region name	Voxel count
Ap Vs nAP	BA 6	1684
Ap Vs nAP	BA 8	440
Ap Vs nAP	BA 20	858
Ap Vs nAP	BA 38	546
Ap Vs nAP	BA 37	266
Ap Vs nAP	BA 47	498
Ap Vs nAP	BA 11	193
mAP Vs nAP	BA 11	89
mAP Vs nAP	BA 5	297
mAP Vs nAP	BA 4	126

*BA = Brodmann area.

Part 2. Supplemental Figure

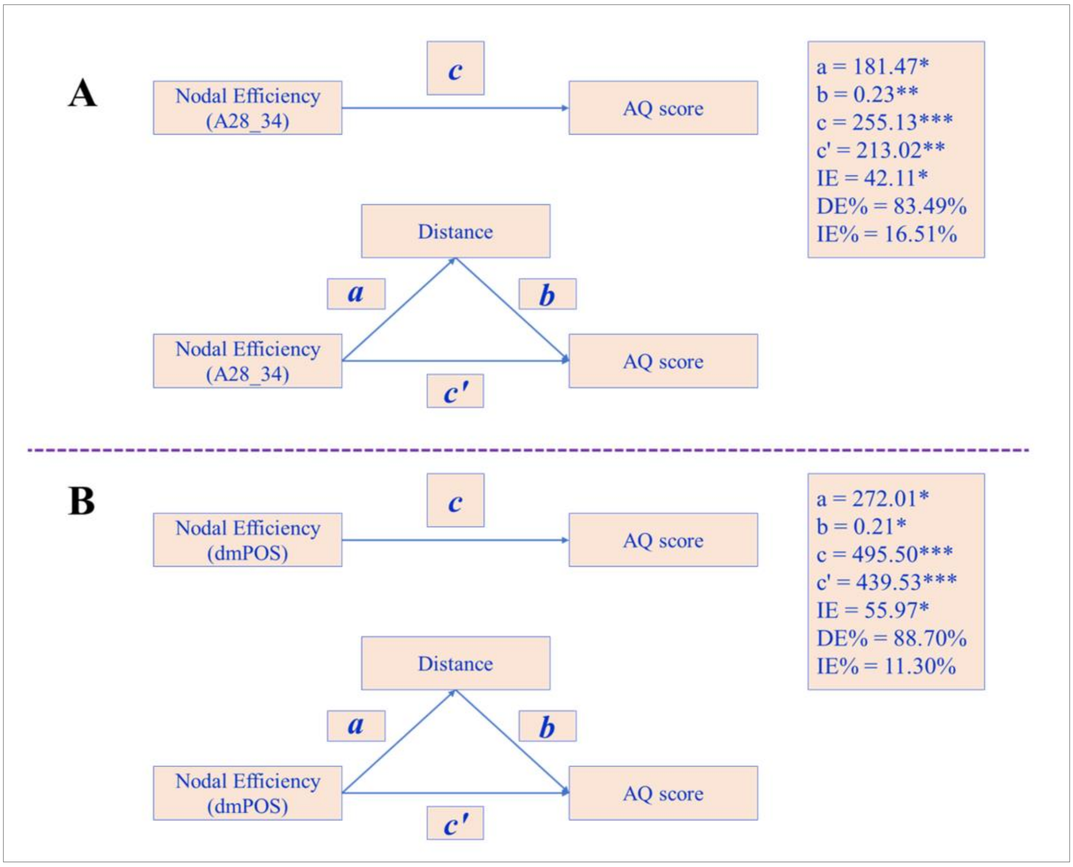

Figure S1. Results of causal mediation. SDTN was a mediator for nodal efficiency of A28_34 and dm_POS to effect on AQ score. A28_34, Brodmann area 28/34 entorhinal cortex; dm_POS, dorsomedial parietal-occipital sulcus.

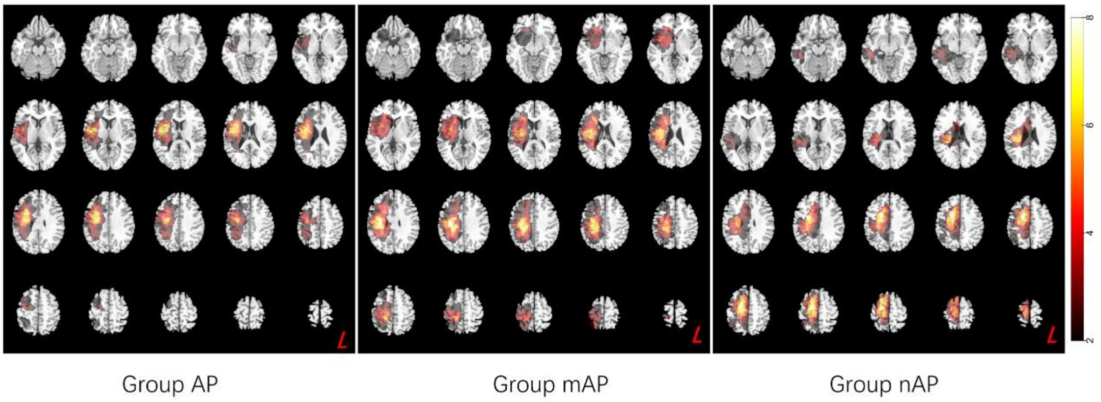

Figure S2: Tumor overlapping map for all three subgroups.The figure shows that the milder the patient's aphasia symptoms, the higher the center of the tumor overlap map, and the further it is from the inferior frontal gyrus, which includes the Broca area.

Part 3. Additional Results on ipisilesional Inferior Frontal Occipital Fasciculus and Uncinate Fasciculus.

Methods and Results

we segmented the IFOF and UF according to JHU-white matter atlas (ipisilateral to tumor), and extracted the mean diffusion metrics of these tracts (Fractional Anisotropy and Mean Diffusivity). One-way ANOVA was used to identify inter-group differences. The figures and tables showing our results are listed below. No significant inter group differences at these tracts was found. But interestingly, from the figure we noticed that some individuals (around 2-4 with in each group) have extra-ordinary high MD and low FA, which strongly indicated robust damage of the corresponding fiber tracts. We believe those may be the tumor-damaged fiber tracts. (Supplementary Table S5 and Figure S3)

Supplementary Table S5: Mean Diffusion metrics of IFOF and UF.

Diffusion Metrics	Value (Mean ± Standard deviation)			One-way ANOVA (*p* value)
	AP	mAP	nAP	
Mean FA of UF	0.385 ± 0.036	0.372 ± 0.062	0.390 ± 0.035	0.403
Mean MD of UF (*10^3)	0.819 ± 0.093	0.858 ± 0.131	0.828 ± 0.109	0.552
Mean FA of IFOF	0.409 ± 0.042	0.381 ± 0.064	0.406 ± 0.031	0.127
Mean MD of IFOF (*10^3)	0.809 ± 0.077	0.846 ± 0.147	0.825 ± 0.091	0.597

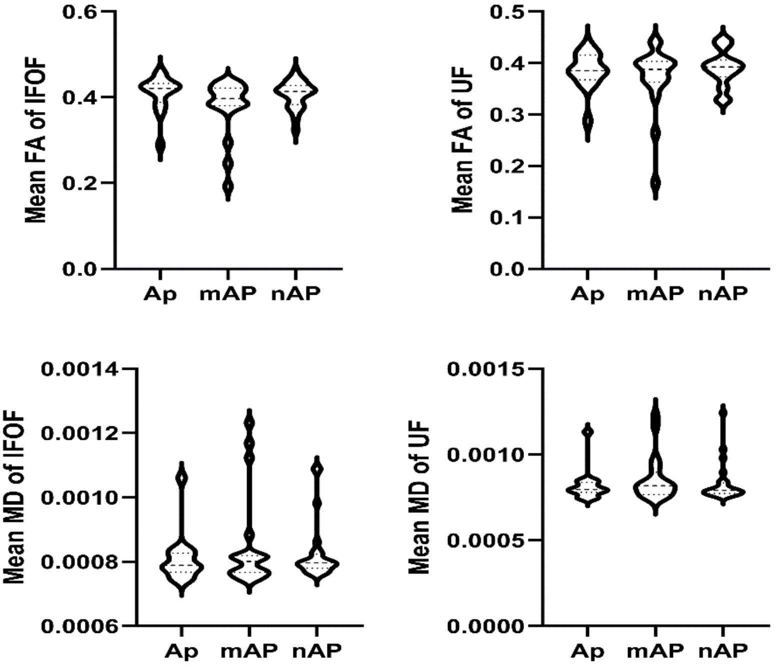

Supplementary Figure S3: Mean Diffusion metrics of IFOF and UF. Statistical analysis showed no inter-group differences. But the figure showed some individuals (around 2-4 with in

each group) have extra-ordinary higher MD and lower FA, which strongly indicated robust damage of the corresponding fiber tracts.
